# Assessment of Bi-Ventricular and Bi-Atrial Areas Using Four-Chamber Cine Cardiovascular Magnetic Resonance Imaging: Fully Automated Segmentation with a U-Net Convolutional Neural Network

**DOI:** 10.3390/ijerph19031401

**Published:** 2022-01-27

**Authors:** Hideo Arai, Masateru Kawakubo, Kenichi Sanui, Ryoji Iwamoto, Hiroshi Nishimura, Toshiaki Kadokami

**Affiliations:** 1 Fukuokaken Saiseikai Futsukaichi Hospital, Chikushino 818-8516, Japan; fukuoka.hideo@gmail.com (H.A.); k-sanui@saiseikai-futsukaichi.org (K.S.); iwamoto_ryouji@kurume-u.ac.jp (R.I.); hnishim3@mac.com (H.N.); t-kadokami@saiseikai-futsukaichi.org (T.K.); 2 Department of Health Sciences, Faculty of Medical Sciences, Kyushu University, Fukuoka 812-8582, Japan

**Keywords:** cardiovascular magnetic resonance imaging, four-chamber cine imaging, fully automatic cardiac segmentation, heart chamber enlargement, convolutional neural network, U-Net

## Abstract

Four-chamber (4CH) cine cardiovascular magnetic resonance imaging (CMR) facilitates simultaneous evaluation of cardiac chambers; however, manual segmentation is time-consuming and subjective in practice. We evaluated deep learning based on a U-Net convolutional neural network (CNN) for fully automated segmentation of the four cardiac chambers using 4CH cine CMR. Cine CMR datasets from patients were randomly assigned for training (1400 images from 70 patients), validation (600 images from 30 patients), and testing (1000 images from 50 patients). We validated manual and automated segmentation based on the U-Net CNN using the dice similarity coefficient (DSC) and Spearman’s rank correlation coefficient (*ρ*); *p* < 0.05 was statistically significant. The overall median DSC showed high similarity (0.89). Automated segmentation correlated strongly with manual segmentation in all chambers—the left and right ventricles, and the left and right atria (end-diastolic area: *ρ* = 0.88, 0.76, 0.92, and 0.87; end-systolic area: *ρ* = 0.81, 0.81, 0.92, and 0.83, respectively; *p* < 0.01). The area under the curve for the left ventricle, left atrium, right ventricle, and right atrium showed high scores (0.96, 0.99, 0.88, and 0.96, respectively). Fully automated segmentation could facilitate simultaneous evaluation and detection of enlargement of the four cardiac chambers without any time-consuming analysis.

## 1. Introduction

Although functional evaluation with medical imaging based on cardiac volume measurement has mainly focused on the left ventricle (LV), its applications in recent research have extended to the left atrium (LA), right ventricle (RV), and right atrium (RA) [1,2,3,4,5,6]. In particular, four-chamber (4CH) imaging with echocardiography has been widely utilized for such measurements because it allows information regarding the four cardiac chambers to be obtained in one cross-sectional image. For example, changes in the left ventricular area are clinically considered as indicators of simplified LV contraction; changes in the right ventricular area are considered as indicators of RV contraction, given that accurate RV volume measurement is difficult due to its complex geometry; and changes in the bilateral atria area are used as indicators of atrial pressure and atrial function [7,8,9,10]. Furthermore, clinical markers that combine the functions of the left and right ventricles and the left and right atria have been proposed in recent years [11,12,13].

Chamber assessment with 4CH echocardiography still has major limitations due to its narrow field of view. On the other hand, cardiac function analysis using cardiovascular magnetic resonance imaging (CMR) allows a relatively wider field of view, has high accuracy, and provides reproducible results; therefore, it has become the gold standard for cardiac function analysis. Recently, independent evaluations have been proposed for each of the four cardiac chambers [14,15]. Although CMR is the gold standard for cardiac function analysis, manual analysis requires considerable time and effort. A high-accuracy analysis support tool, such as feature tracking, has been proposed; however, its efficacy in terms of time reduction is limited. Moreover, observer-dependent errors owing to the subjectivity of the analysis using semiautomatic feature-tracking measurements cannot be eliminated. To solve these problems, highly accurate automatic extraction of the LV and RV using deep learning for short-axis cine CMR images has been reported [16,17]. However, to the best of our knowledge, there are no reports of automatic extraction of cardiac chambers using 4CH cine CMR. We hypothesized that it would be clinically useful to automatically measure the areas of the four cardiac chambers with cine CMR images. This study aimed to investigate the accuracy of automatic extraction of the four cardiac areas using deep learning with 4CH cine CMR and verify the effectiveness of this approach.

## 2. Materials and Methods

### 2.1. Cine CMR Datasets

For this study, 150 consecutive patients (age: 68 ± 12 years; men: 96; women: 54) with known or suspected cardiac dysfunction, were between July 2018 and September 2020 retrospectively enrolled, and their 4CH cine CMR images of a middle slice of the heart were used for evaluation. Patients who presented unacceptable imaging artifacts due to magnetic inhomogeneity or motion were excluded from the study. All datasets were randomly assigned to groups for training (1400 images from 70 patients), validation (600 images from 30 patients), and testing (1000 images from 50 patients). This retrospective observational study was approved by the institutional review board of Futsukaichi Hospital (approval number 201) and Kyushu University (approval number 29-199) and conducted in accordance with the 1964 Declaration of Helsinki. Because the requirement for obtaining patient consent was waived for this study, an online provision on the hospital homepage was prospectively made available to the patients, allowing them to opt out of the study.

### 2.2. CMR Parameters

Cine CMR was performed using a 3.0-T magnetic resonance imaging (MRI) system (Ingenia, Philips Healthcare, Best, The Netherlands) equipped with a 33-mT/m maximum gradient strength, 120-T/ms slew rate, and a 32-channel phased-array receiver coil. An electrocardiography-gated steady-state free precession cine image was acquired in the 4CH view with 20 reconstructed phases per heartbeat. The cine sequence parameters were as follows: repetition time/echo time, 2.9/1.47 ms; flip angle, 50°; slice thickness, 6 mm; field of view, 350 × 350 mm^2^; acquisition matrix, 176 × 208; reconstruction matrix, 352 × 352; and SENSE (sensitivity encoding) factor, 2.5.

### 2.3. Image Pre-Processing

The matrix size for the deep learning algorithm was downsized from 352 × 352 to 128 × 128 with nearest neighbour interpolation. Subsequently, the bit depth for all the datasets were converted from 16 bits to 8 bits of portable network graphics files. The window level and width were rescaled to 256 gradients with the minimum and maximum values for each image intensity. These imaging processes were performed using an offline software (MATLAB (R2017b; MathWorks, Inc., Natick, MA, USA)).

### 2.4. Image Segmentation Using Manual Delineation

Delineation of the endocardium of the four cardiac chambers for a cardiac cycle was performed by adopting a concerted measure by analysts with 10–15 years of clinical experience in cardiac radiology. The papillary muscles and trabeculations were included in the blood pool. Delineation was performed using the MATLAB software [18].

### 2.5. Image Segmentation Using Deep Learning

To develop an established CNN, we used a deep learning platform called Neural Network Console (Sony Network Communications, Tokyo, Japan) and a specialized graphic processing unit (GeForce GTX 1080; Nvidia, Santa Clara, CA, USA) [19]. In general, data augmentation is performed based on the following parameters: rotation range, ±5.0°; flip vertical. Consequently, the segmentation models for bi-ventricular and bi-atrial areas were generated using the 100-epoch U-Net architecture with a batch size of 10, learning rate of 0.002, momentum optimizer, and weight decay of 0.0005 [20].

### 2.6. Image Post-Processing

Each image predicted by the developed U-Net CNN contained blood pools that do not correspond to each cardiac chamber. To correct the morphology, the predicted images underwent morphological closing using a 3-pixel radius disk, based on selection of the largest area and as reported previously [17]. The closed images were then binarized by the adapted threshold method [21]. These imaging processes were performed using MATLAB.

### 2.7. Evaluation Criteria

The performance of segmentation was evaluated using the Dice similarity coefficient (*DSC*) [22]. The *DSC* is described by Equation (1), where *X* represents the manually drawn contour and *Y* represents the prediction produced by the U-Net CNN.

(1) $$DSC=\frac{2\left|X\;\cap \;Y\right|}{\left|X\right|+\left|Y\right|}$$

Further, the performance of the segmentation was evaluated using the Mahalanobis distance (*MD*) [23]. The *MD* is described by Equation (2), where *X* represents the manually drawn contour and *Y* represents the prediction by U-Net CNN. The factor *T* represents the matrix transpose, and *S*^−1^ represents the inverse of the pooled covariance matrix of the two images; this matrix is computed as the weighted average of the two covariance matrices.

(2) $$MD^{2}={\left(X^{-}-Y^{-}\right)}^{T}S^{-1}\left(X^{-}-Y^{-}\right)$$

### 2.8. Image Categorization

Test datasets comprised 20 consecutive images per patient through an entire cardiac cycle. The ventricle with the smallest area and the atrium with the largest area were defined as the end-systolic area (ESA), and its first phase was defined as the end-diastolic area (EDA). The fractional area change (FAC) was calculated as the percentage of the difference between the EDA and ESA with respect to EDA.

### 2.9. Statistical Analyses

The Shapiro–Wilk test was used to assess the normality of the data distribution. Due to non-normal data distribution, descriptive statistics data were presented as the median and corresponding interquartile ranges. The correlations between the area in each cardiac chamber from both manual segmentation and automated segmentation and based on the U-Net CNN were analysed using Spearman’s rank correlation coefficient (*ρ*). The accuracy of the U-Net CNN with respect to the manual method was evaluated using Bland–Altman analysis and the intraclass correlation coefficient (ICC) with one-way random single measures. The ICC values were defined as excellent (ICC ≥ 0.75), good (ICC = 0.60–0.74), moderate (ICC = 0.40–0.59), and poor (ICC ≤ 0.39). Receiver operating characteristic (ROC) analysis was performed to assess the accuracy of detection of heart chamber enlargement using the U-Net CNN-derived EDA. Chamber enlargement was diagnosed based on the value reported in previous studies [24,25]. Diagnostic cut-off values of chamber enlargement as left ventricular EDV/body surface area (BSA), left atrial EDA/BSA, right ventricular EDA/BSA, and right atrial EDA/BSA were (men: 108 mL/m^2^; women: 96 mL/m^2^), (men: 11.9 cm^2^/m^2^; women: 12.7 cm^2^/m^2^), (men: 13.6 cm^2^/m^2^; women: 12.6 cm^2^/m^2^), and (men: 11.1 cm^2^/m^2^; women: 11.0 cm^2^/m^2^), respectively. All statistical analyses were performed using GraphPad Prism version. 9.0.1, for Windows (GraphPad Software, San Diego, CA, USA). Statistical significance was set at *p* < 0.05.

## 3. Results

The patients’ characteristics are shown in Table 1. The clinical characteristics were compared between the training, validation, and test groups. For comparison, one-way analysis of variance or the Kruskal–Wallis test was used depending on the distribution of the data. There were no significant differences among the groups in the results of the comparisons.

### 3.1. Accuracy of Segmentation of the Four Cardiac Chambers

**Figure 1:**
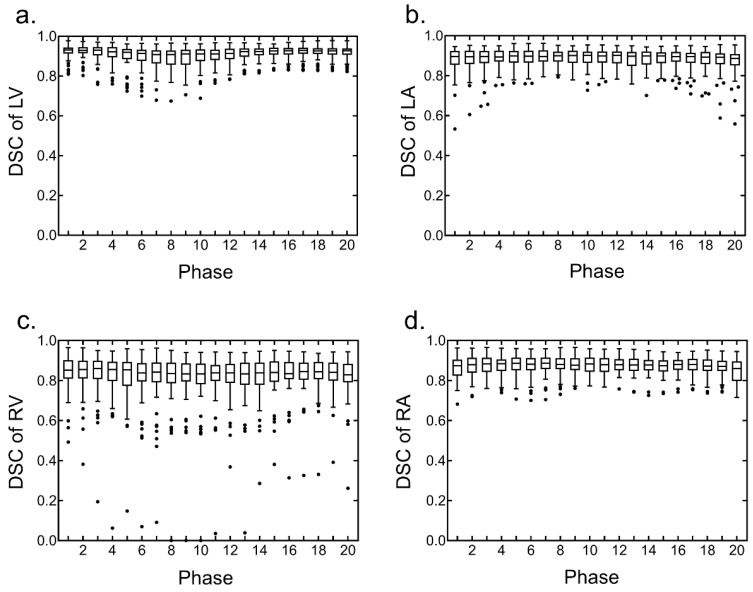
Distribution of Dice similarity coefficient (DSC) for the four cardiac chamber segmentations using the U-Net convolutional neural network (CNN) on the test datasets. Box plots were generated by using GraphPad Prism for all cardiac phases in each chamber. (**a**) DSC of the left ventricle (LV), (**b**) DSC of the left atrium (LA), (**c**) DSC of the right ventricle (RV), and (**d**) DSC of the right atrium (RA).

**Figure 2:**
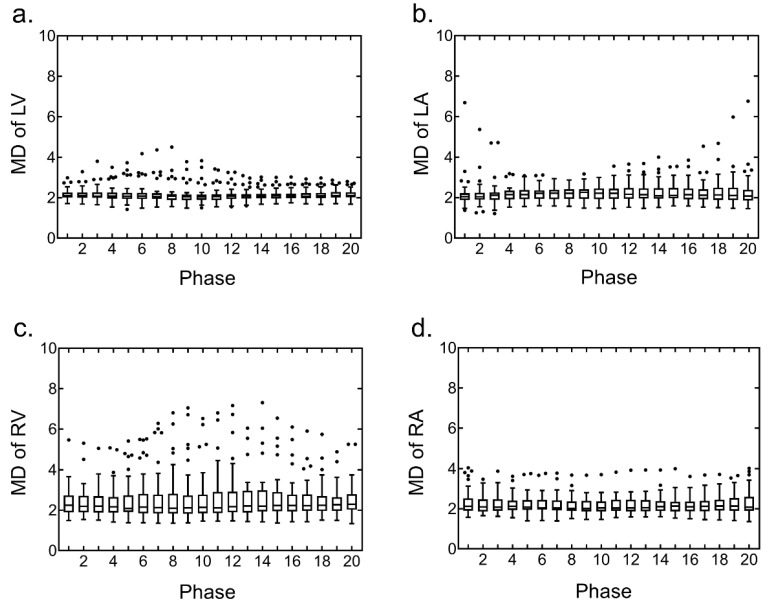
Distribution of the Mahalanobis distance (MD) for the four cardiac chamber segmentations using U-Net convolutional neural network (CNN) on the test datasets. Box plots were generated using GraphPad Prism for all cardiac phases in each chamber. (**a**) MD of the left ventricle (LV), (**b**) MD of the left atrium (LA), (**c**) MD of the right ventricle (RV), and (**d**) MD of the right atrium (RA).

The segmentation of the four cardiac chambers using the U-Net CNN was able to detect all the chambers in the test datasets. The segmentation accuracy of the four cardiac chambers was evaluated between images obtained with manual segmentation and automated segmentation using the U-Net CNN; the overall median DSC was 0.89, and the median DSCs in each chamber were 0.92, 0.90, 0.84, and 0.88 in the LV, LA, RV, and RA, respectively. In each chamber, the DSC for all the cardiac phases demonstrated high values (Figure 1). The segmentation accuracy of the four cardiac chambers images obtained with manual segmentation and automated segmentation using U-Net CNN was evaluated; the overall median MD was 2.11, and the median MDs in each chamber were 2.08, 2.14, 2.18, and 2.07 in the LV, LA, RV, and RA, respectively. In each chamber, the MD for all the cardiac phases demonstrated low values (Figure 2).

**Figure 3:**
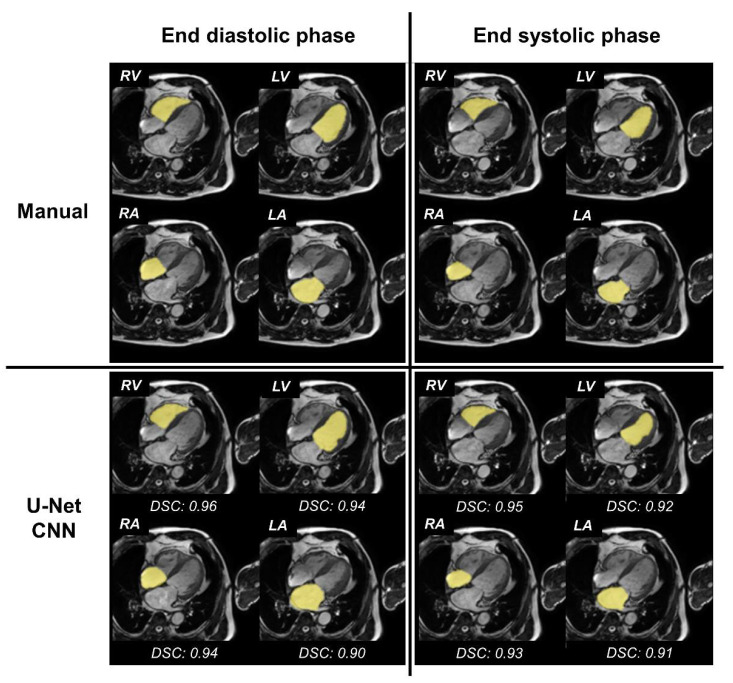
Four cardiac chamber segmentation—results of a representative case. Upper row: fusion images with both original image of four-chamber (4CH) cine cardiovascular magnetic resonance imaging (CMR) and label images extracted by the manual method. Lower row: fusion images with both the original image of 4CH cine CMR and label images extracted by the fully automated method with U-Net convolutional neural network (CNN). Left column: fusion images at the end-diastolic phase. Right column: fusion images at the end-systolic phase. RV = right ventricle; LV = left ventricle; RA = right atrium; LA = left atrium; DSC = Dice similarity coefficient.

We have illustrated the segmentation results of the four cardiac chambers in Figure 3 for representative cases.

### 3.2. Functional Parameters of the Four Cardiac Chambers

**Figure 4:**
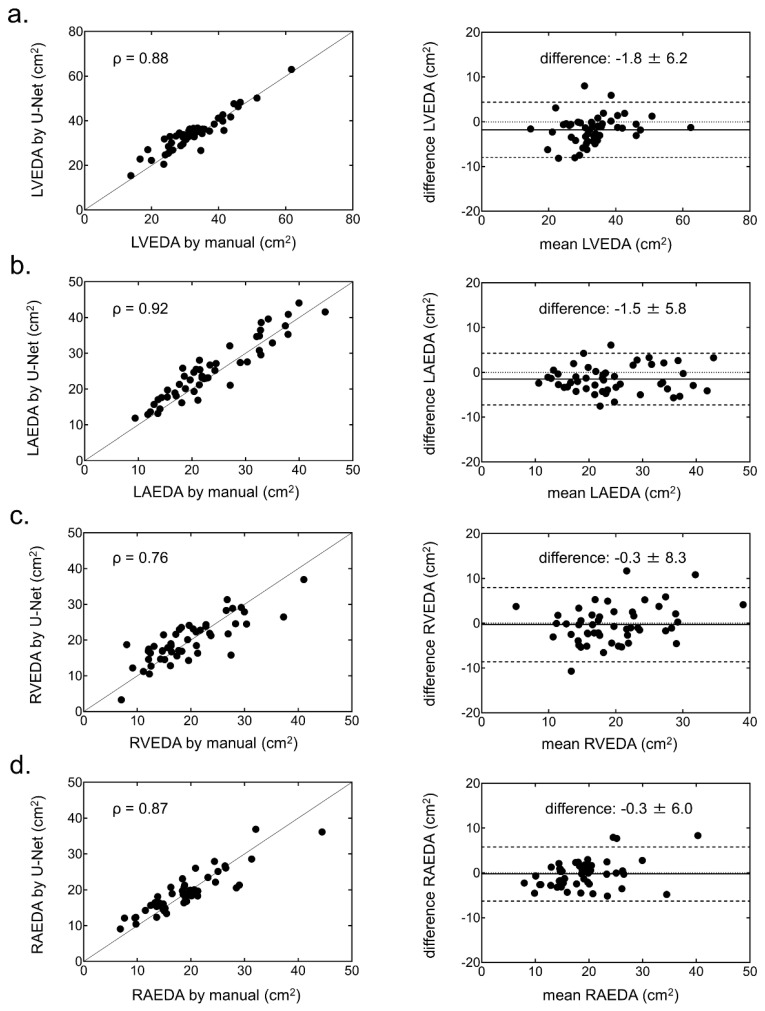
Correlation (left) and Bland-Altman (right) plots of the end-diastolic area (EDA) in each chamber generated from the manual method and the U-Net CNN. (**a**) Left ventricular (LV) EDA, (**b**) left atrial (LA) EDA, (**c**) right ventricular (RV) EDA, and (**d**) right atrial (RA) EDA.

**Figure 5:**
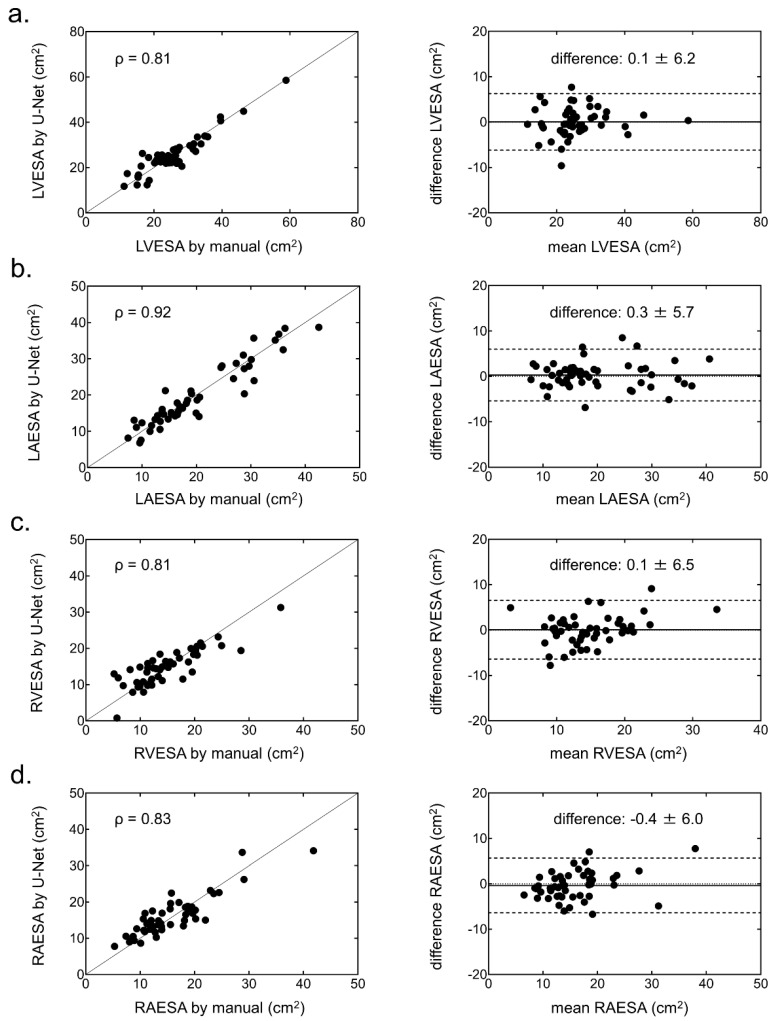
Correlation (left) and Bland-Altman (right) plots of the end-systolic area (ESA) in each chamber generated from the manual method and U-Net CNN. (**a**) Left ventricular (LV) ESA, (**b**) left atrial (LA) ESA, (**c**) right ventricular (RV) ESA, and (**d**) right atrial (RA) ESA.

The EDA and ESA with the U-Net CNN in each chamber showed strong correlations and small biases against the EDA and ESA with manual segmentation (Figure 4 and Figure 5).

**Figure 6:**
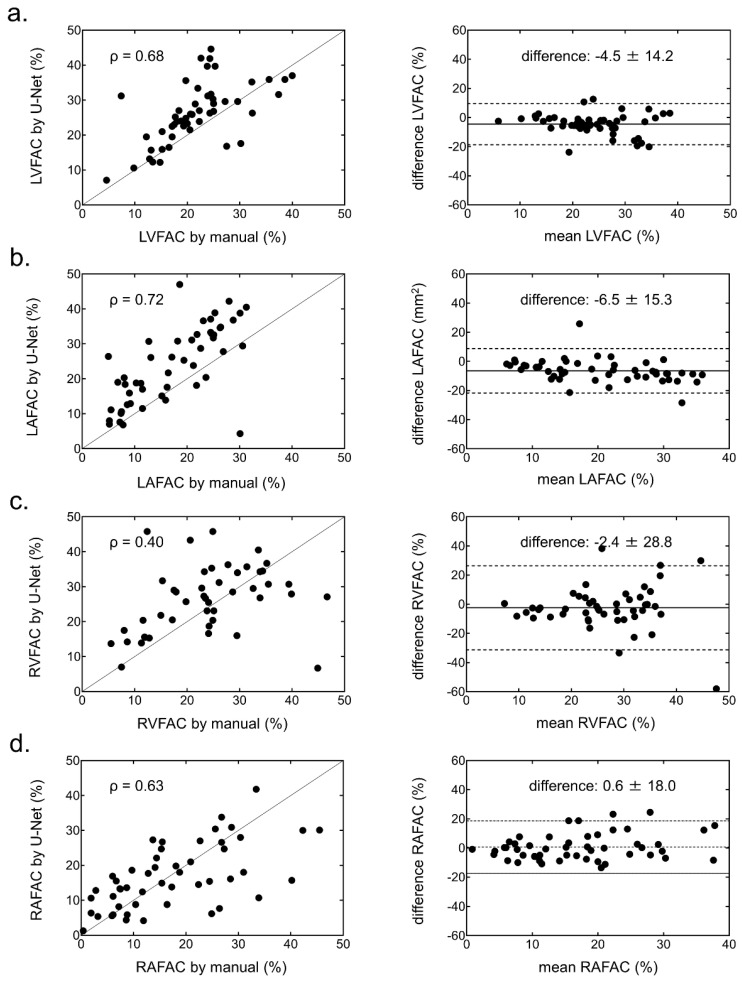
Correlation (left) and Bland-Altman (right) plots of fractional area change (FAC) in each chamber generated from the manual method and U-Net CNN. (**a**) Left ventricular (LV) FAC, (**b**) left atrial (LA) FAC, (**c**) right ventricular (RV) FAC, and (**d**) right atrial (RA) FAC.

The biases of FAC in each chamber were small (Figure 6), and the FAC in the LV and LA showed strong correlation with the U-Net CNN. However, the correlation of FAC in the RV and RA was weaker than that in the left side of the heart.

**Figure 7:**
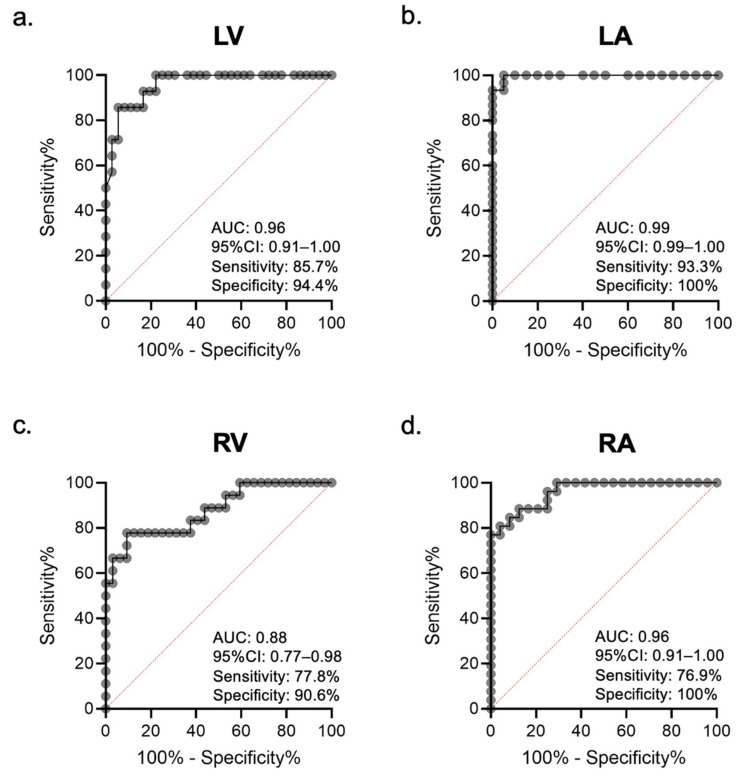
Receiver operating characteristic (ROC) curves of the chamber area extracted by the U-Net convolutional neural network (CNN) for identifying a patient with an enlargement of each heart chamber. (**a**) Left ventricle (LV), (**b**) left atrium (LA), (**c**) right ventricle (RV), and (**d**) right atrium (RA). AUC = area under the curve.

The ICCs of the EDA and ESA in the LV, LA, RV, and RA showed excellent variability, as highlighted by their respective values (EDA: 0.93, 0.94, 0.80, and 0.88; ESA: 0.93, 0.94, 0.82, and 0.87). The ICCs of FAC in the LV, LA, and RA showed good variability (0.61, 0.68, and 0.60, respectively), except it was poor in the RV only (0.19). ROC analysis revealed optimal EDA values to identify cardiac chamber enlargement (23.8, 12.9, 13.4, and 11.2 cm^2^/m^2^ in the LV, LA, RV, and RA, respectively). The area under the curves and the 95% confidence intervals of the LV, LA, RV, and RA were 0.96 (0.91–1.00), 0.99 (0.99–1.00), 0.88 (0.77–0.98), and 0.96 (0.91–1.00), respectively (Figure 7).

### 3.3. Computation Time

The time of supervised learning was linearly dependent on the number of training and validation images and the number of epochs. The average time of learning with 12,000 images and 20 epochs was 26 min. Once trained, the U-Net CNN was able to perform fully automated segmentation of 4CH cine CMR at approximately 0.02 s per cine image (20 s per 1000 images).

## 4. Discussion

We evaluated the accuracy of segmentation with 4CH cine CMR using the U-Net CNN to test the datasets of 50 patients. Our results revealed high similarity and correlation for all the cardiac phases in each chamber. Once the U-Net CNN learns to train datasets, it can perform fast and accurate segmentation without a visually dependent manual method. In addition, accurate fully automated segmentation of the four cardiac chambers allowed automatic detection of the presence or absence of four cardiac chamber enlargement without any subjective measurement and time-consuming analysis.

In this study, we performed four cardiac chamber segmentations in all cardiac phase images of cine CMR using U-Net CNN. The DSC of all cardiac phases in each chamber showed high similarity without any variations caused by the different phases. Thus, U-Net CNN can perform four cardiac chamber segmentations independent of the cardiac phase. In our study, LV segmentation revealed a high correlation, similar to that reported previously [16,26]. One possible explanation for this could be that the LV structure comprises a thick myocardium with a simple morphology. The area correlations of the cardiac chambers, excluding LV, also exhibited high values. These automatically extracted values obtained using fully automated segmentation have the potential for useful clinical application. However, FAC, a contraction indicator calculated based on the areas, exhibited a low correlation on the right side of the heart. The right side of the heart has a more complex morphology than the left side; it has a considerably thinner myocardium and undergoes significant movement in the direction of the long axis. These factors can make myocardial extraction difficult and prevent accurate extraction of the cardiac chambers. Thus, we believe that accumulation of slight errors on the right side of the heart resulted in a weak correlation of the FAC on the right side in our study. Similar to that reported previously, the low extraction accuracy of the right side of the heart remains a major challenge for automated segmentation based on U-Net CNN, and further work is required in this field [17,26,27,28]. Nevertheless, we demonstrated that U-Net CNN enables extraction of areas in all the four cardiac chambers, with high accuracy, using 4CH cine CMR images. Our study suggests that fully automated segmentation areas using U-Net CNN can be employed for clinical applications.

Some studies have recently demonstrated the accuracy of segmentation of cine CMR using the U-Net CNN [16,26]. Many of these studies have reported on the LV using short-axis cine CMR. The accuracy of LV segmentation has been high in these studies, similar to our results. A few studies have demonstrated the accuracy of RV segmentation, although the performance of RV segmentation was lower than that of LV segmentation [27,28]. In traditional segmentations with short axis cine CMR, it was necessary for the observers to visually define the analytical slices for each chamber to determine the scope of analysis. In other words, the above studies have used automated segmentation under limited conditions, and this remains a major challenge in understanding fully automated segmentation. In contrast, it was not necessary for the observers in our study to visually define the analytical slices; moreover, fully automated segmentations of EDA and ESA for each of the four cardiac chambers could be performed. These segmentations allowed extraction of the chambers and detection of heart chamber enlargement without any subjective measurement and time-consuming analysis. Figure 5 accurately indicates the detection of heart chamber enlargement in each of the four chambers. Compared to the short axis cine CMR, 4CH cine CMR has the ability to evaluate all the heart chambers. In our study, automated heart chamber extraction was able to detect heart chamber enlargement due to pressure or volume overload. Future improvements in the accuracy of heart chamber extraction may lead to strain analysis that is useful in assessing local wall movements or heart chamber synchronization.

One limitation of our study is that it was performed at a single centre with a limited patient population. However, as a preliminary study to validate the use of four cardiac chamber segmentations using the U-Net CNN, the results of our study can still be useful. Further refinement of segmentation requires additional research in the near future, such as the use of other CNN architectures and the use of larger datasets.

## 5. Conclusions

We developed and evaluated a fully automated 4CH cine CMR segmentation method based on a U-Net CNN. Our automated method was able to extract each chamber in 4CH cine CMR with high accuracy, and without any time-consuming and subjective analysis, and detect heart chamber enlargement.

## Figures and Tables

**Table 1:** Patient characteristics.

	Training (*n* = 70)	Validation (*n* = 30)	Test (*n* = 50)	*p* Value
**Clinical c** **haracteristics**				
Age, years	66 ± 13	71 ± 10	68 ± 12	0.20
Male	48 (69)	19 (63)	29 (58)	0.49
Heart rate, beats/min	65 ± 13	65 ± 13	70 ± 16	0.27
Weight, kg	64 ± 13	60 ± 13	60 ± 14	0.16
Height, cm	163 ± 9	162 ± 9	161 ± 10	0.48
Body surface area, m^2^	1.7 ± 0.2	1.6 ± 0.2	1.6 ± 0.3	0.16
Body mass index, kg	24 ± 4	23 ± 3	23 ± 4	0.42
**Cardiovascular disease**				
ICM	10 (14)	12 (40)	10 (20)	
HHD	6 (9)	1 (3)	3 (6)	
Arrhythmia	18 (26)	9 (30)	14 (28)	
HCM	9 (13)	3 (10)	9 (18)	
DCM	8 (11)	1 (3)	3 (6)	
Sarcoidosis	3 (4)	1 (3)	2 (4)	
Amyloidosis	7 (10)	2 (7)	2 (4)	
Others	9 (13)	1 (3)	7 (14)	
**CMR measurements**				
**LV function**				
EDV, mL	169 ± 86	137 ± 40	143 ± 51	0.25
ESV, mL	110 ± 116	70 ± 41	77 ± 48	0.25
EF, %	46 ± 17	52 ± 16	49 ± 14	0.28

Baseline characteristics of all subjects are shown. Values are mean ± SD or *n* (%). Training datasets include validation datasets. ICM = ischemic cardiomyopathy; HHD = hypertensive heart disease; HCM = hypertrophic cardiomyopathy; DCM = dilated cardiomyopathy; CMR = cardiac magnetic resonance; LV = left ventricle; EDV = end-diastolic volume; ESV = end-systolic volume; EF = ejection fraction; SD, standard deviation.

## Data Availability

The data presented in this study are available on request from the authors (M.K. and H.A.).
